# The Urobiomes of Adult Women With Various Lower Urinary Tract Symptoms Status Differ: A Re-Analysis

**DOI:** 10.3389/fcimb.2022.860408

**Published:** 2022-06-09

**Authors:** Cara Joyce, Thomas Halverson, Caroline Gonzalez, Linda Brubaker, Alan J. Wolfe

**Affiliations:** ^1^ Dept. of Medicine, Stritch School of Medicine, Loyola University Chicago, Maywood, IL, United States; ^2^ Dept. of Microbiology and Immunology, Stritch School of Medicine, Loyola University Chicago, Maywood, IL, United States; ^3^ Dept. of Obstetrics, Gynecology and Reproductive Sciences, University of California San Diego, La Jolla, CA, United States

**Keywords:** urinary microbiome, lower urinary symptom, enhanced culture, reanalysed data, women’s health

## Abstract

The discovery of the urinary microbiome (urobiome) has created opportunities for urinary health researchers who study a wide variety of human health conditions. This manuscript describes an analysis of catheterized urine samples obtained from 1,004 urobiome study participants with the goal of identifying the most abundant and/or prevalent (common) taxa in five clinically relevant cohorts: unaffected adult women (n=346, 34.6%), urgency urinary incontinence (UUI) (n=255, 25.5%), stress urinary incontinence (SUI) (n=50, 5.0%), urinary tract infection (UTI) (n=304, 30.4%), and interstitial cystitis/painful bladder syndrome (IC/PBS) (n=49, 4.9%). Urine was collected *via* transurethral catheter and assessed for microbes with the Expanded Quantitative Urine Culture (EQUC) technique. For this combined analytic cohort, the mean age was 59 ± 16; most were Caucasian (n=704, 70.2%), Black (n=137, 13.7%), or Hispanic (n=130, 13.0%), and the mean BMI was 30.4 ± 7.7. Whereas many control or IC/PBS cohort members were EQUC-negative (42.4% and 39.8%, respectively), members of the other 3 cohorts were extremely likely to have detectable microbes. The detected urobiomes of the controls and IC/PBS did not differ by alpha diversity or genus level composition and differed by only a few species. The other 3 cohorts differed significantly from the controls. As expected, *Escherichia* was both prevalent and highly abundant in the UTI cohort, but other taxa also were prevalent at more moderate abundances, including members of the genera *Lactobacillus, Streptococcus, Staphylococcus, Corynebacterium*, *Actinomyces*, and *Aerococcus.* Members of these genera were also prevalent and highly abundant in members of the UUI cohort, especially *Streptococcus anginosus*. Intriguingly, these taxa were also detected in controls but at vastly lower levels of both prevalence and abundance, suggesting the possibility that UUI-associated symptoms could be the result of an overabundance of typical urobiome constituents. Finally, prevalence and abundance of microbes in the SUI cohort were intermediate to those of the UUI and control cohorts.

These observations can inform the next decade of urobiome research, with the goal of clarifying the mechanisms of urobiome community composition and function. There is tremendous potential to improve diagnosis, evaluation and treatment for individuals affected with a wide variety of urinary tract disorders.

## Introduction

The discovery of the urinary microbiome has created opportunities for urinary health researchers in a wide variety of human health conditions. The first decade of urobiome studies have revealed clear associations between various microbes and various urinary symptoms and diagnoses. Several species have been associated with urgency urinary incontinence, including *Actinotignum* (previously *Actinobaculum*) *schaalii*, *Streptococcus anginosus*, and *Aerococcus urinae* (Pearce et al., 2014). Some evidence supports the hypothesis that *A. urinae* is also associated with urinary tract infection; since standard urine culture often misses *A. urinae* (Price et al., 2016), this and other evidence induces doubts concerning diagnoses of exclusion, such as UUI. Some women with lower urinary tract symptoms have urobiomes predominated by supposedly beneficial taxa, especially *Lactobacillus* species (Pearce et al., 2014; Pearce et al., 2015; Thomas-White et al., 2018). Finally, some evidence exists associating pre-treatment urobiome status with response to medical UUI treatment with an anticholinergic medication (Thomas-White et al., 2016) and response to surgical treatment with midurethral sling for mixed urinary incontinence (Richter et al., 2022).

However, most early studies were small with sample sizes in the few dozens and were designed to only identify associations. As we begin the second decade of urobiome research, studies with increased samples sizes will be needed to verify and test these associations and their underlying mechanisms. To inform those efforts, this manuscript describes an analysis of catheterized urine samples obtained from 1,004 urobiome study participants (both unpublished and previously published). Our goal was to characterize the urobiome of women with lower urinary tract symptoms and identify the most abundant and/or prevalent (common) taxa in five clinically relevant cohorts. These cohorts were unaffected adult women, as well as those affected by common lower urinary tract conditions, including urinary tract infection (UTI), interstitial cystitis/painful bladder syndrome (IC/PBS), and two common forms of urinary incontinence: urgency urinary incontinence (UUI) and stress urinary incontinence (SUI). This descriptive analysis may inform future prospective work that incorporates knowledge of the urobiome composition in diagnosing and treating women presenting with symptoms.

## Methods

### Recruitment of Participants

The urobiome of 1004 participants previously reported in eight published studies were reanalyzed using five cohorts of clinical interest: UTI (n=304, 30.4%), UUI (n=255, 25.5%), SUI (n=50, 5.0%), IC/PBS (n=49, 4.9%), and controls (n=346, 34.6%) without these lower urinary tract symptoms. The inclusion criteria for each cohort were based on predominant presenting symptoms/diagnoses as assessed by the relevant validated questionnaire (see original publications). These participants were pooled from 8 separate, IRB-approved studies with identical baseline assessment and sample collection procedures. 921 of these participants were at least partially described previously (**Supplemental Table 1**). These studies were supported by NIDDK (R01DK104718-01A1, R56DK104718-01, R21DK097435, and P20DK108268), a PFD Research Grant and a grant from the Falk Foundation (LU202567). The funders played no part in the design or conduct of the study.

### Urine Collection and Analysis

Urine was collected aseptically *via* transurethral catheter, according to standard clinical protocols, and placed in a BD Vacutainer Plus C&S preservative tube. To detect microbes, Expanded Quantitative Urine Culture (EQUC) was used, as described (Hilt et al., 2014). Briefly, 0.1 mL of urine was spread quantitatively onto BAP, Chocolate and Colistin, Naladixic Acid (CNA) agars (BD BBL™ Prepared Plated Media), then incubated in 5% CO_2_ (35°C for 48 hours). A second set of BAPs were inoculated with 0.1 mL of urine and incubated in room atmosphere at 35°C and 30°C for 48 hours, respectively. In addition, 0.1 mL of urine was inoculated onto each of two CDC Anaerobe 5% sheep blood agar (ABAP) plates (BD BBL™ Prepared Plated Media) and incubated in either a Campy gas mixture (5% O_2_, 10% CO_2_, 85% N) or in anaerobic conditions at 35°C for 48 hours. The detection level was 10 CFU/mL, represented by 1 colony of growth on any of the plates. Each morphologically distinct colony type was isolated on a different plate of the same media to prepare a pure culture that was used for microbe identification. Matrix-Assisted Laser Desorption Ionization Time of Flight Mass Spectrometry (MALDI-TOF MS) with the MALDI Biotyper 3.0 software (Bruker Daltonics, Billerica, MA) was used to identify the bacterial isolates.

### Statistical Analysis

Generalized linear models were used to calculate age-adjusted estimates of microbial abundance, prevalence, and diversity for each cohort. Omnibus tests for cohort differences were assessed for each model, and group comparisons were reported as significant when Sidak-corrected p-values were less than 0.05. Alpha diversity measures included the unique number of genera (richness), Pielou’s index (evenness), the Shannon index (richness and evenness), and Simpson index (richness and abundance). Each alpha diversity measure was regressed on age and cohort in a separate general linear model with normal distribution and identity link. Alpha diversity measures were also correlated with age and body mass index, and Spearman’s rho with 95% confidence intervals were estimated for each cohort. For each genus, abundance (mean CFU/mL) was modeled as a negative binomial distributed variable and regressed on cohort and age. Prior to modeling, mean CFU/mL for any taxon was calculated by dividing the total number of CFU/mL by the number of plates with growth for that taxon. The prevalence of each genus or species was defined as the proportion of individuals with any CFU/mL detected, and logistic regression analyses were conducted to estimate the adjusted mean prevalence for each cohort. The probability of no growth was modeled in a separate age-adjusted logistic regression analysis. SAS version 9.4 was used for statistical modeling, and microbiome diversity measure calculations and data visualization were performed using R version 4.0.3.

## Results

The demographics of each study-specific cohort were described within the original studies. For this combined analytic cohort, the mean age was 59 ± 16, most were Caucasian (n=704, 70.2%), Black (n=137, 13.7%), or Hispanic (n=130, 13.0%), and mean BMI was 30.4 ± 7.7. The UTI and UUI cohorts were 10-15 years older on average than the other 3 cohorts. Participants in the control and IC/PBS cohorts were most likely to have no growth by EQUC: 42.4% and 39.8% of each cohort, respectively. In contrast, growth was not detected in only 5.6%, 7.9%, and 0.5% of the UTI, UUI and SUI cohorts, respectively (**Table 1**).

**Table 1:** Participant characteristics.

	UTI	UUI	SUI	IC/PBS	Control
N	304	255	50	49	346
Age, mean (SD) [n=1001]	66 (16)	65 (12)	54 (14)	51 (16)	50 (14)
Body mass index (kg/m²), mean (SD) [n=914]	29.8 (7.2)	31.8 (8.5)	30.2 (6.9)	--	29.9 (7.5)
Race/ethnicity, n (%) [n=1003]					
Non-Hispanic White	224 (73.7)	182 (71.4)	36 (72.0)	33 (67.3)	229 (66.4)
Non-Hispanic Black	34 (11.2)	42 (16.5)	3 (6.0)	1 (2.0)	57 (16.5)
Hispanic	39 (12.8)	23 (9.1)	8 (16.0)	13 (26.5)	47 (13.6)
Other	7 (2.3)	8 (3.1)	3 (6.0)	2 (4.1)	12 (3.5)
EQUC-negative (%)	5.6	7.9	0.5	39.8	42.4

After adjusting for age, the detected urobiomes of the UTI and control cohorts differed in the number of genera (a measure of richness) and Simpson’s Index (which measures richness and abundance) but not Peilou’s evenness or Shannon’s Index (which measures both richness and evenness) (**Table 2**). The UUI and controls differed in number of genera, Shannon’s Index, and Peilou’s evenness, but not Simpson’s Index. The SUI and control cohorts differed in all 4 measures of alpha diversity, whereas the IC/PBS and control cohorts were similar. The UTI and UUI cohorts differed in all 4 measures, while the 2 incontinent cohorts (UUI and SUI) differed in the number of species and Shannon’s Index (**Table 2**). Correlations of age and body mass index with diversity indices were small to moderate across cohorts (**Supplemental Table 2**). Older age was associated with higher diversity indices in the UUI and SUI cohorts and lower diversity indices for UTI. Higher BMI was associated with a greater number of genera detected across cohorts, and higher Shannon and Simpson indices in the UTI cohort.

**Table 2:** Age-adjusted diversity indices.

	UTI	UUI	SUI	IC/PBS	Control
Number of genera	2.3 ± 0.1^be^	5.1 ± 0.1^acde^	2.3 ± 0.3^be^	1.6 ± 0.3^b^	1.3 ± 0.1^abc^
Pielou’s evenness*	0.22 ± 0.02^bc^	0.51 ± 0.02^ae^	0.48 ± 0.05^ae^	0.35 ± 0.06	0.23 ± 0.02^bc^
Shannon index	0.16 ± 0.03^bc^	0.77 ± 0.03^acde^	0.39 ± 0.06^abe^	0.33 ± 0.06^b^	0.19 ± 0.02^bc^
Simpson index	0.17 ± 0.02^bcde^	0.47 ± 0.02^a^	0.34 ± 0.05^ade^	0.58 ± 0.05^ac^	0.54 ± 0.02^ac^

^a^Different from UTI; ^b^different from UUI; ^c^different from SUI; ^d^different from IC/PBS; ^e^different from Control. *calculated among those with at least two genera.

**Figure 1:**
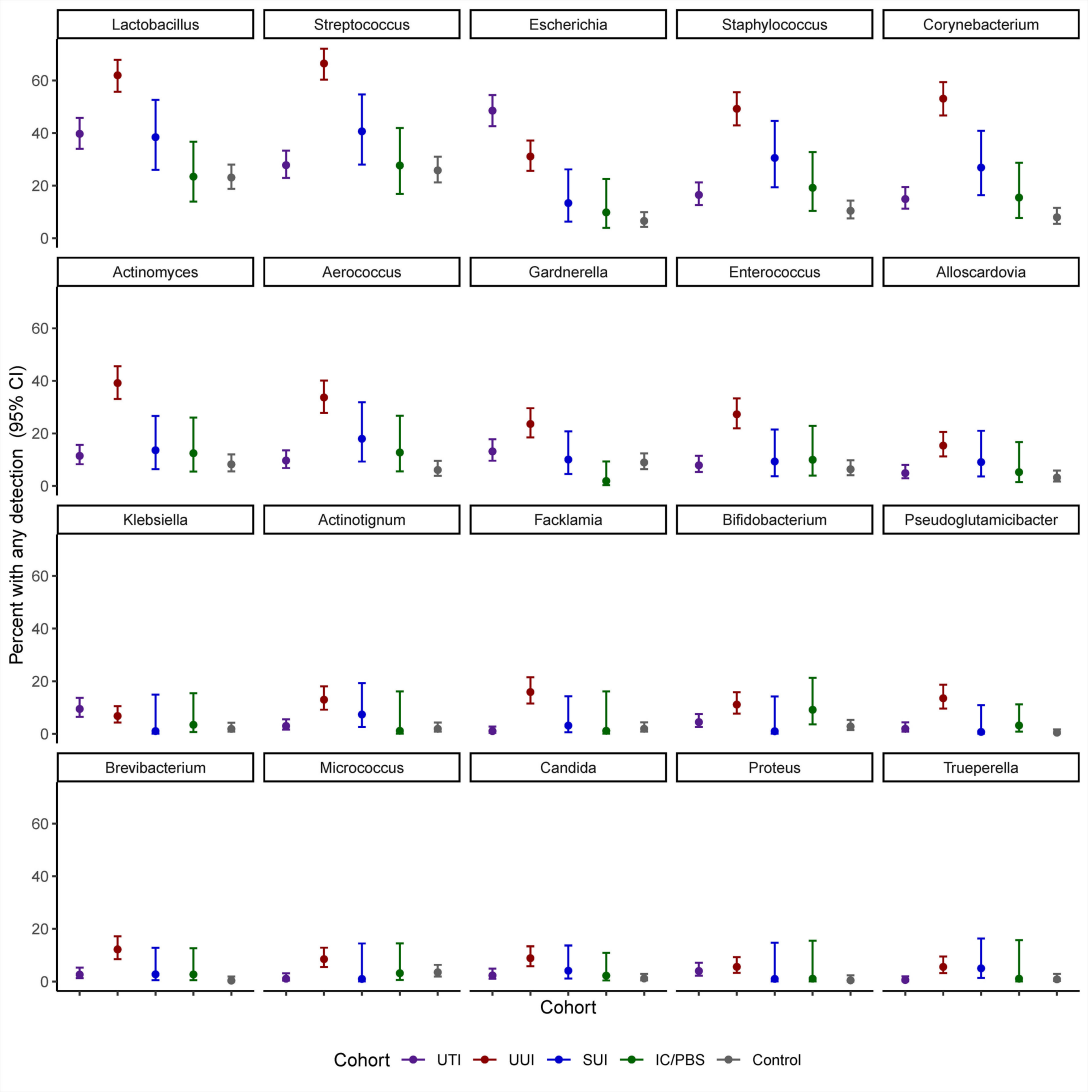
Age adjusted prevalence (%) with 95% confidence interval by cohort.

Of the detected genera, 20 were present in at least 2% of participants and investigated in models of prevalence by cohort (**Figure 1** and **Table 3**). *Escherichia* was more prevalent in the UTI cohort (48.5%) than all others, but several other genera were detected in more than 10% of the UTI members, including *Lactobacillus* (39.7%)*, Streptococcus* (27.8%), *Staphylococcus* (16.5%), *Corynebacterium* (14.9%), *Gardnerella* (13.2%), and *Actinomyces* (11.5%). More genera were commonly detected in the UUI cohort, with over 20% adjusted prevalence of each of the most prevalent genera in the UTI cohort plus *Enterococcus.* Among those with SUI, *Lactobacillus, Streptococcus*, and *Staphylococcus* were the genera most often detected. Prevalence was generally lower in the IC/PBS and control cohorts across all genera, with *Lactobacillus* and *Streptococcus* the most common of those detected.

**Table 3:** Age adjusted prevalence (%) ± standard error.

	UTI	UUI	SUI	IC/PBS	Control
Lactobacillus	39.7 + 3.0^be^	62.0 + 3.1^acde^	38.4 + 6.9^b^	23.4 + 5.9^b^	23.1 + 2.4^ab^
Streptococcus	27.8 + 2.7^b^	66.5 + 3.0^acde^	40.7 + 7.0^b^	27.7 + 6.5^b^	25.8 + 2.5^b^
Escherichia	48.5 + 3.0^bcde^	31.1 + 3.0^ae^	13.4 + 4.9^a^	9.8 + 4.4^a^	6.6 + 1.4^ab^
Staphylococcus	16.5 + 2.2^b^	49.2 + 3.2^ade^	30.5 + 6.5^e^	19.2 + 5.7^b^	10.4 + 1.7^bc^
Corynebacterium	14.9 + 2.1^b^	53.1 + 3.2^acde^	26.9 + 6.3^be^	15.5 + 5.3^b^	8.0 + 1.5^bc^
Actinomyces	11.5 + 1.9^b^	39.2 + 3.2^acde^	13.6 + 5.0^b^	12.5 + 5.0^b^	8.3 + 1.6^b^
Aerococcus	9.7 + 1.7^b^	33.7 + 3.1^ae^	18.0 + 5.7	12.8 + 5.2	6.1 + 1.4^b^
Gardnerella	13.2 + 2.1^b^	23.6 + 2.8^ade^	10.1 + 3.9	1.9 + 1.6^b^	9.0 + 1.5^b^
Enterococcus	7.9 + 1.6^b^	27.3 + 2.9^ae^	9.3 + 4.2	10.0 + 4.5	6.4 + 1.4^b^
Alloscardovia	4.9 + 1.3^b^	15.4 + 2.4^ae^	9.1 + 4.1	5.3 + 3.3	3.2 + 1.0^b^
Klebsiella	9.5 + 1.8^e^	6.8 + 1.6	1.0 + 1.5	3.4 + 2.8	1.9 + 0.8^a^
Actinotignum	3.0 + 0.9^b^	13.0 + 2.2^ae^	7.4 + 3.8	1.1 + 1.6	1.9 + 0.8^b^
Facklamia	1.0 + 0.5^b^	15.9 + 2.5^ae^	3.1 + 2.5	1.2 + 1.6	1.9 + 0.8^b^
Bifidobacterium	4.4 + 1.2^b^	11.1 + 2.0^ae^	1.0 + 1.4	9.2 + 4.2	2.8 + 0.9^b^
Pseudoglutamicibacter	1.9 + 0.8^b^	13.5 + 2.3^ae^	0.7 + 1.0	3.2 + 2.1	0.5 + 0.3^b^
Brevibacterium	2.7 + 1.0^b^	12.2 + 2.2^ae^	2.8 + 2.3	2.7 + 2.2	0.4 + 0.3^b^
Micrococcus	1.1 + 0.6^b^	8.6 + 1.8^a^	1.0 + 1.4	3.2 + 2.6	3.6 + 1.1
Candida	2.4 + 0.9^b^	8.9 + 1.9^ae^	4.1 + 2.6	2.3 + 1.9	1.2 + 0.5^b^
Proteus	4.0 + 1.2	5.6 + 1.5^e^	1.0 + 1.5	1.1 + 1.6	0.5 + 0.4^b^
Trueperella	0.6 + 0.4^b^	5.6 + 1.6^a^	5.1 + 3.2	1.1 + 1.6	0.9 + 0.5
No growth	5.6 + 1.4^de^	7.9 + 1.7^de^	10.5 + 4.3^de^	39.8 + 7.1^abc^	42.2 + 2.9^abc^

^a^Different from UTI; ^b^different from UUI; ^c^different from SUI; ^d^different from IC/PBS; ^e^different from Control.

**Figure 2:**
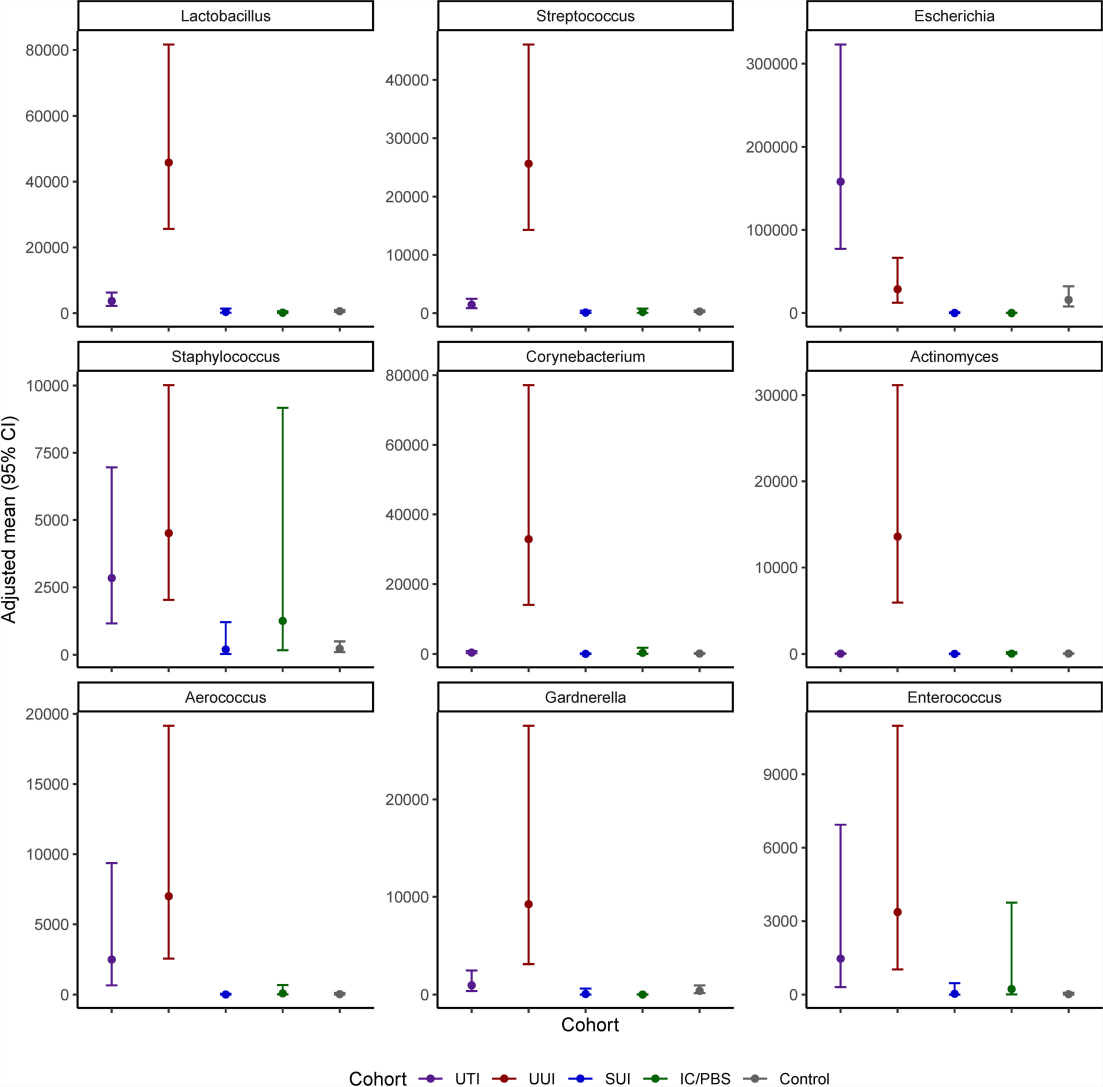
Age adjusted mean abundance (CFU/mL) with 95% confidence interval by cohort.

Nine genera were both prevalent in at least 10% of participants across cohorts and exhibited significant cohort differences in abundance: *Lactobacillus, Streptococcus, Escherichia, Staphylococcus, Corynebacterium, Actinomyces, Aerococcus, Gardnerella*, and *Enterococcus* (p<0.05 for all omnibus tests of cohort differences) (**Figure 2** and **Table 4**). *Corynebacterium*, *Lactobacillus, Streptococcus, Actinomyces* and *Gardnerella* were significantly more abundant (generally 2 orders of magnitude) in participants with UUI than those in the non-UTI cohorts; these genera were more abundant in the UUI than the UTI cohort, except that the difference was generally 1 order of magnitude. *Corynebacterium* and *Actinomyces* were the notable exceptions, being 2 orders of magnitude more abundant in the UUI cohort than the UTI cohort. *Aerococcus* was significantly more abundant in the UUI cohort compared to the non-UTI cohorts. *Escherichia* was significantly more abundant in those with UTI compared to UUI, SUI, IC/PBS, and controls, but it was considerably more abundant in UUI and controls relative to SUI and IC/PBS. *Staphylococcus* was more abundant in the UTI and UUI cohorts compared to the other cohorts.

**Table 4:** Age adjusted mean (CFU/mL) ± standard error.

	UTI	UUI	SUI	IC/PBS	Control
Lactobacillus	3703 + 999^bcde^	45774 + 13531^acde^	379 + 252^ab^	149 + 100 ^ab^	580 + 148 ^ab^
Streptococcus	1458 + 396^bce^	25640 + 7671^acde^	118 + 79^ab^	217 + 145^b^	279 + 72^ab^
Escherichia	158063 + 57687^bcde^	28559 + 12304^acd^	137 + 124^abe^	57 + 53^abe^	15871 + 5706^acd^
Staphylococcus	2843 + 1299^e^	4513 + 1836^ce^	197 + 182^b^	1250 + 1271	223 + 90^ab^
Corynebacterium	388 + 141^bc^	32892 + 14307^acde^	25 + 22^ab^	317 + 279^b^	105 + 35^b^
Actinomyces	32 + 12^b^	13587 + 5756^acde^	5 + 5^b^	32 + 30^b^	38 + 13^b^
Aerococcus	2484 + 1682^ce^	6998 + 3595^cde^	8 + 8^ab^	90 + 93^b^	26 + 21^ab^
Gardnerella	953 + 463^bd^	9251 + 5152^acde^	59 + 71^b^	2 + 2^abe^	378 + 176^bd^
Enterococcus	1463 + 1162^e^	3367 + 2031^ce^	35 + 47^b^	228 + 326	19 + 14^ab^

^a^Different from UTI; ^b^different from UUI; ^c^different from SUI; ^d^different from IC/PBS; ^e^different from Control.

Of the nine prevalent genera with differential abundances, 6 included multiple species: *Lactobacillus, Streptococcus, Staphylococcus, Corynebacterium, Actinomyces*, and *Aerococcus* (**Table 5**). Most species in these genera were differentially more prevalent in the UUI cohort than the controls: 4 of 5 *Lactobacillus* species, 2 of 6 *Streptococcus* species, 5 of 7 *Staphylococcus* species, 9 of 9 *Corynebacterium* species, 2 of 3 *Actinomyces* species, and both *Aerococcus* species. *Streptococcus anginosus* merits special mention; it was more prevalent in the UUI cohort relative to all the other cohorts, being detected in 49% of that cohort. Another noteworthy species was *Staphylococcus epidermidis*, which was more prevalent in the UUI, SUI and IC/PBS cohorts relative to the controls.

**Table 5:** Age adjusted prevalence (%) ± standard error.

	UTI	UUI	SUI	IC/PBS	Control
Lactobacillus					
crispatus	8.1 ± 1.6	15.1 ± 2.4^e^	10.3 ± 4.9	4.9 ± 2.7	5.7 ± 1.2^b^
gasseri	14.5 ± 2.1^b^	29.6 ± 3.6^ae^	12.9 ± 4.8	11.2 ± 4.6	9.6 ± 1.7^b^
iners	10.9 ± 1.9^b^	21.4 ± 2.8^ae^	15.3 ± 4.8	8.7 ± 3.4	6.8 ± 1.3^b^
jensenii	9.6 ± 1.8	18.0 ± 2.5^e^	14.2 ± 4.7	3.7 ± 2.3	6.0 ± 1.2^b^
rhamnosus	2.4 ± 3.9	3.2 ± 1.1	1.1 ± 1.5	1.2 ± 1.6	0.5 ± 3.4
Streptococcus					
agalactiae	8.7 ± 1.7	13.1 ± 2.2^e^	10.1 ± 4.2	9.8 ± 4.1	4.7 ± 1.1^b^
anginosus	16.4 ± 2.2^b^	49.3 ± 3.2^acde^	26.4 ± 6.2^be^	10.9 ± 4.5^b^	11.4 ± 1.8^bc^
mitis	3.7 ± 1.1^b^	9.9 ± 1.9^a^	7.0 ± 3.6	3.1 ± 2.5	4.6 ± 1.2
oralis	2.3 ± 6.8^bd^	8.8 ± 1.8^a^	7.3 ± 3.8	12.3 ± 5.5^a^	4.8 ± 1.3
parasanguinis	0.6 ± 5.4^bc^	4.8 ± 1.3^a^	7.4 ± 3.8^a^	5.8 ± 3.6	3.6 ± 1.1
salivarius	0.7 ± 0.4^b^	5.4 ± 1.4^a^	5.3 ± 3.3	1.1 ± 1.6	3.9 ± 1.1
Staphylococcus					
aureus	2.0 ± 7.8	4.0 ± 1.3	9.2 ± 4.2^e^	1.1 ± 1.5	1.4 ± 1.7^c^
capitis	1.2 ± 0.6	5.4 ± 1.5	3.0 ± 2.4	1.0 ± 1.4	1.3 ± 2.6
epidermidis	7.4 ± 1.5^b^	36.8 ± 3.2^ae^	18.8 ± 5.6^e^	17.2 ± 5.5^e^	4.3 ± 1.1^bcd^
haemolyticus	2.0 ± 2.8^b^	21.3 ± 2.8^ae^	5.1 ± 3.2	5.3 ± 3.3	1.0 ± 9.6^b^
hominis	2.8 ± 1.8^b^	19.3 ± 2.6^ae^	2.9 ± 2.4	6.9 ± 3.6	1.5 ± 7.7^b^
lugdunensis	2.1 ± 5.9^b^	8.4 ± 1.8^ae^	3.0 ± 2.4	7.2 ± 3.8^e^	0.4 ± 5.4^bd^
simulans	1.6 ± 9.7^b^	10.2 ± 2.2^ae^	5.2 ± 3.2	3.3 ± 2.6	1.1 ± 1.6^b^
Corynebacterium					
amycolatum	4.3 ± 1.2^b^	31.0 ± 3.1^acde^	3.0 ± 2.4^b^	7.2 ± 3.8^b^	1.9 ± 6.8^b^
aurimucosum	4.0 ± 1.2^b^	24.5 ± 2.9^ae^	10.4 ± 4.3^e^	4.7 ± 3.1	1.7 ± 7.7^bc^
coyleae	5.1 ± 1.3^b^	29.9 ± 3.3^ace^	8.8 ± 4.6^b^	11.1 ± 4.5	2.7 ± 8.9^b^
Imitans	2.9 ± 1.7^b^	12.1 ± 2.2^ae^	2.8 ± 2.3	4.7 ± 3.1	0.6 ± 8.4^b^
lipophile group	1.5 ± 9.7^b^	11.3 ± 2.1^ae^	7.3 ± 3.8^e^	3.4 ± 2.7	0.4 ± 9.4^bc^
Riegelii	0.7 ± 6.5^b^	11.7 ± 2.2^ae^	3.1 ± 2.5	3.3 ± 2.6	1.1 ± 2.6^b^
tuberculostearic	1.1 ± 2.6^b^	7.0 ± 1.7^ae^	1.0 ± 1.4	5.4 ± 3.3	0.7 ± 9.5^b^
tuscaniense	0.8 ± 6.5^b^	9.3 ± 2.9^ae^	1.0 ± 1.4	3.0 ± 2.5	0.4 ± 4.4^b^
urealyticum	0.3 ± 6.3^b^	6.2 ± 1.7^ae^	1.1 ± 1.5	1.2 ± 1.6	0.5 ± 3.4^b^
Actinomyces					
europaeus	1.3 ± 9.6^b^	6.3 ± 1.6^a^	1.1 ± 1.5	3.5 ± 2.8	1.5 ± 6.7^b^
neuii	6.2 ± 1.4^b^	23.4 ± 2.8^ae^	11.5 ± 4.7	5.6 ± 3.5	3.5 ± 1.1^b^
turicensis	2.7 ± 6.9^b^	13.7 ± 2.3^ae^	3.1 ± 2.5	5.8 ± 3.6	3.3 ± 1.1^b^
Aerococcus					
sanguinicola	2.8 ± 0.9^b^	12.4 ± 2.2^ae^	9.5 ± 4.3	1.1 ± 1.6	0.1 ± 7.2^b^
urinae	7.2 ± 1.4^b^	29.8 ± 3.1^ae^	15.7 ± 5.4	10.4 ± 4.8	4.4 ± 1.2^b^

^a^Different from UTI; ^b^different from UUI; ^c^different from SUI; ^d^different from IC/PBS; ^e^different from Control.

Of all those species, only 9 were differentially abundant (*Streptococcus anginosus, Lactobacillus gasseri, Aerococcus urinae, Staphylococcus epidermidis, Lactobacillus iners, Corynebacterium coyleae, Actinomyces neuii, Lactobacillus jensenii*, and *Corynebacterium amycolatum*) (**Table 6**). Relative to the controls, 6 were more abundant in the UTI cohort, whereas all 9 were significantly more abundant in the UUI cohort. Again, *S. anginosus* merits notice; it was almost 10 and 100 times more abundant in the UTI and UUI cohorts, respectively. Whereas all 3 of the *Lactobacillus* species were more abundant in the UUI cohort, only *L. gasseri* was more abundant in the UTI cohort. *A. urinae* was more abundant in the UTI, UTI and IC/PBS cohorts, *S. epidermidis* was more abundant in the UTI and UUI cohorts, and *A. neuii* was more abundant in the UUI cohort. Whereas *C. amycolatum* was more abundant in the UTI, UUI, and IC/PBS cohorts, *C. coyleae* was more abundant in the UUI cohort but less abundant in the UTI cohort.

**Table 6:** Age adjusted mean (CFU/mL) ± standard error

	UTI	UUI	SUI	IC/PBS	Control
Streptococcus anginosus	697 ± 258^bce^	10098 ± 3976^acde^	33 ± 29^ab^	131 ± 122^b^	84 ± 29^ab^
Lactobacillus gasseri	1034 ± 480^bce^	7782 ± 3674^acde^	10 ± 11^ab^	97 ± 104^b^	28 ± 12^ab^
Lactobacillus iners	384 ± 192^bd^	9655 ± 5150^acde^	155 ± 189^b^	8 ± 10^ab^	272 ± 125^b^
Lactobacillus jensenii	1105 ± 661	12724 ± 8098^cde^	23 ± 35^b^	18 ± 27^b^	224 ± 129^b^
Aerococcus urinae	564 ± 253^bce^	4127 ± 1853^acde^	4 ± 4^ab^	66 ± 68^be^	1 ± 1^abd^
Staphylococcus epidermidis	3489 ± 2488^ce^	4845 ± 2839^cde^	2 ± 3^ab^	51 ± 62^b^	20 ± 15^ab^
Actinomyces neuii	10 ± 5^b^	8872 ± 5010^acde^	4 ± 6^b^	3 ± 4^b^	31 ± 15^b^
Corynebacterium amycolatum	208 ± 118^bce^	12808 ± 8598^acde^	0 ± 0^abd^	30 ± 38^bce^	0 ± 0^abd^
Corynebacterium coyleae	7 ± 4^be^	5802 ± 3385^acde^	1 ± 2^be^	104 ± 132^b^	176 ± 84^abc^

^a^Different from UTI; ^b^different from UUI; ^c^different from SUI; ^d^different from IC/PBS; ^e^different from Control.

## Discussion

Amongst these five cohorts of clinical interest, we detected group differences in urobiome characteristics. However, despite these group differences, the cohorts have between-group overlap that precludes accurate predictions of diagnostic categorization. In addition, within group variation suggests that a range of urobiome characteristics cannot be associated with a single diagnostic cohort. This is consistent with the evolving idea that many lower urinary tract symptoms have heterogenous etiologies and/or mechanisms. Nonetheless, the trends observed can be used to inform the second decade of urobiome research, with the goal of clarifying the mechanisms of urobiome community function and membership. We are hopeful that future, well-designed longitudinal research will contribute valuable insights.

The etiology of painful bladder syndrome and interstitial cystitis continues to challenge clinicians and researchers who hope to find a way to ease the chronic suffering of affected patients. Although many hoped to find a single causative microbe, the “single microbe” hope has not been supported by research to date. Women with IC/PBS were just as likely to be EQUC-negative as healthy controls. Of those with detectable urobiomes, there was no difference in alpha diversity or composition at the genus level, and only a few differences in either prevalence or abundance but not both at the species level. Although there are clear and profound clinical differences between these two cohorts (Jacobs et al., 2021), these findings suggest that there is not likely to be a major microbial etiology.

This analysis clearly demonstrates that, compared to the urobiomes of controls, richness was increased in the urobiome of the UUI, SUI and UTI cohorts, with the most richness found in the UUI cohort. While the UUI and SUI cohorts were richer, more even, and more abundant than the controls, the UUI cohort was richer than the SUI cohort and the UUI cohort was richer, more even and more abundant than the UTI cohort. Both forms of incontinence are chronic conditions, with likely longer term, chronic changes in the urobiome. Especially in women who are seeking treatment for incontinence, the effect of evaluation and treatment may also affect the urobiome that was sampled in these patients. In contrast, UTI is typically episodic. While there is little research into microbial recovery following UTI, it is expected that the urobiome attempts to return to the “normal” state, ideally closer to the urobiome seen in the control cohort.

Within the UUI cohort, we detected multiple genera and species, many of which were prevalent and abundant. Many are of uncertain clinical significance and await further research to clarify the role of the individual microbe and its role in the function of the urobiome it inhabits. However, our findings that *Aerococcus urinae* is abundant in both UTI and UUI cohorts is not expected. Since *A. urinae* is often not detected on standard urine culture (Price et al., 2016), this finding supports the use of enhanced microbial detection methods in women with UUI, such as expanded culture techniques, to detect and appropriately treat this known uropathogen. Without appropriate detection, it is likely that symptomatic women would be deemed “infection free”; subsequent diagnosis and treatment would focus on a UUI diagnosis with the missed opportunity to treat a known uropathogen. *A. urinae-*associated UUI may be an important, treatable subset of UUI patients. This testable hypothesis should be addressed as a research priority as soon as feasible.

We observed the expected disproportionate prevalence and abundance of *Escherichia* in the UTI cohort. *Escherichia* was less prevalent in the UUI and control cohorts but at relatively high abundance when detected. Other genera differed across cohorts. For example, while *Lactobacillus* is generally considered to be beneficial, it is likely that the beneficial contributions are determined at the species level and possible that certain *Lactobacillus* species (and other presumed beneficial microbes) can be opportunistic uropathogens within a given urobiome. For example, our finding that *L. gasseri*, *L. iners*, and other presumed commensal/beneficial genera often detected in controls are more abundant in the UUI cohort may suggest the possibility that the urobiome of these individuals could be affected by overgrowth of commensal/beneficial genera, a suggestion made more than 30 years ago by Rosalind Maskell and her team (Wilkins et al., 1989). Other detected species are thought to be emerging uropathogens and have been reported to be associated with UUI (e.g., *S. anginosus* and *A. neuii* [recently renamed *Winkia neuii* (Nouioui et al., 2018)]).

This analysis benefits from multiple strengths, most notably the relatively large numbers once the five individual cohorts were pooled. Also, the urine samples were obtained by transurethral catheterization, increasing the likelihood that the urine is of bladder origin. Additionally, the use of EQUC ensures that living microbes are being assessed. Furthermore, all cohorts were characterized with well validated instruments for the specific lower urinary tract condition of interest. Finally, the results were adjusted for age, given the current evidence that age itself may affect microbial niches.

Limitations include knowledge that EQUC does not detect every bacterial taxon and is especially limited for strict anaerobes. It also does not detect viruses or most eukaryotic microbes. Thus, the list of genera and species is likely incomplete. Also, given the design of the initial studies, this analysis cannot provide insights into longitudinal changes for individuals or across cohorts. Future prospective longitudinal studies should consider change in the urobiome composition as relates to diagnosis, symptom severity, treatment options, response, and recurrence. Finally, current clinical diagnostic categories were adopted prior to discovery of the urobiome; these categories may require revision as research closes important knowledge gaps.

In conclusion, as we enter the second decade of urobiome research, there is tremendous potential to improve diagnosis, evaluation and treatment for individuals affected with a wide variety of urinary tract disorders. As the community of urobiome researchers expands, covering the entire urinary tract and exploring both benign and malignant disease, we expect that significant advances will rapidly occur. The next decade of research is also likely to benefit from less expensive microbiome technology, a larger pool of knowledgeable researchers who collaborate in multi-disciplinary teams, and an increased opportunity to acquire research funds.

## Data Availability Statement

The data analyzed in this study is subject to the following licenses/restrictions: This is a reanalysis. The data are present in the original papers. Requests to access these datasets should be directed to awolfe@luc.edu.

## Ethics Statement

The studies involving human participants were reviewed and approved by Loyola University Chicago IRB. The patients/participants provided their written informed consent to participate in this study.

## Author Contributions

CJ: Data Analysis, manuscript writing/editing. TH: Project development, data collection, data analysis, manuscript review. CG: Data collection, manuscript review. LB: Project development, manuscript writing/editing. AW: Project development, manuscript writing/editing. All authors contributed to the article and approved the submitted version.

## Funding

These studies were supported by NIDDK (R01DK104718-01A1, R56DK104718-01, R21DK097435, and P20DK108268), a PFD Research Grant and a grant from the Falk Foundation (LU202567). The funders played no part in the design or conduct of the study.

## Conflict of Interest

The authors declare that the research was conducted in the absence of any commercial or financial relationships that could be construed as a potential conflict of interest.

## Publisher’s Note

All claims expressed in this article are solely those of the authors and do not necessarily represent those of their affiliated organizations, or those of the publisher, the editors and the reviewers. Any product that may be evaluated in this article, or claim that may be made by its manufacturer, is not guaranteed or endorsed by the publisher.

## Supplementary Material

Supplemental Table 1 Source of participants for this pooled analysis. (1) 111 peri-menopausal women with UUI prior to treatment with solifenacin or asymptomatic controls, (2) 154 women with UTI-like symptoms or asymptomatic controls, (3) 134 peri-menopausal asymptomatic controls, (4) 89 women with IC/PBS or asymptomatic controls, (5) 145 women with SUI, UUI, or asymptomatic controls, (6) 63 postmenopausal women with UUI prior to receipt of vaginal estrogen therapy, (7) 225 women with symptoms of UTI recruited to a trial evaluating the expanded urine culture protocol, and (8) 83 peri-menopausal women with UUI prior to oral treatment with mirabegron.

Supplemental Table 2 Correlation of participant characteristics with diversity indices. Spearman’s correlation coefficient calculated with 95% confidence interval separately for each cohort and each alpha diversity index.

## References

[B1] Hilt E. E. McKinley K. Pearce M. M. Rosenfeld A. B. Zilliox M. J. Mueller E. R.. (2014). Urine is Not Sterile: Use of Enhanced Urine Culture Techniques to Detect Resident Bacterial Flora in the Adult Female Bladder. J. Clin. Microbiol. 52, 871–876. doi: 10.1128/JCM.02876-13 24371246 PMC3957746

[B2] Jacobs K. M. Price T. K. Thomas-White K. Halverson T. Davies A. Myers D. L.. (2021). Cultivable Bacteria in Urine of Women With Interstitial Cystitis: (Not) What We Expected. Female. Pelvic. Med. Reconstr. Surg. 27, 322–327. doi: 10.1097/SPV.0000000000000854 32265402 PMC8088819

[B3] Nouioui I. Carro L. Garcia-Lopez M. Meier-Kolthoff J. P. Woyke T. Kyrpides N. C.. (2018). Genome-Based Taxonomic Classification of the Phylum Actinobacteria. Front. Microbiol. 9. doi: 10.3389/fmicb.2018.02007 PMC6113628 30186281

[B4] Pearce M. M. Hilt E. E. Rosenfeld A. B. Zilliox M. J. Thomas-White K. Fok C.. (2014). The Female Urinary Microbiome: A Comparison of Women With and Without Urgency Urinary Incontinence. MBio 5, e01283–e01214. doi: 10.1128/mBio.01283-14 25006228 PMC4161260

[B5] Pearce M. M. Zilliox M. J. Rosenfeld A. B. Thomas-White K. J. Richter H. E. Nager C. W.. (2015) And Network Pelvic Floor Disorders. 2015. The Female Urinary Microbiome in Urgency Urinary Incontinence. Am. J. Obstet. Gynecol. (2015) 213, 347 e1–347 11. doi: 10.1016/j.ajog.2015.07.009 26210757 PMC4556587

[B6] Price T. K. Dune T. Hilt E. E. Thomas-White K. J. Kliethermes S. Brincat C.. (2016). The Clinical Urine Culture: Enhanced Techniques Improve Detection of Clinically Relevant Microorganisms. J. Clin. Microbiol. 54, 1216–1222. doi: 10.1128/JCM.00044-16 26962083 PMC4844725

[B7] Richter H. E. Carnes M. U. Komesu Y. M. Lukacz E. S. Arya L. Bradley M.. (2022). Health Eunice Kennedy Shriver National Institute of Child, and Network Human Development Pelvic Floor Disorders. 2022. Association Between the Urogenital Microbiome and Surgical Treatment Response in Women Undergoing Midurethral Sling Operation for Mixed Urinary Incontinence. Am. J. Obstet. Gynecol. (2022) 226, 93 e1–93 e15. doi: 10.1016/j.ajog.2021.07.008 34297969 PMC8748268

[B8] Thomas-White K. J. Gao X. Lin H. Fok C. S. Ghanayem K. Mueller E. R.. (2018). Urinary Microbes and Postoperative Urinary Tract Infection Risk in Urogynecologic Surgical Patients. Int. Urogynecol. J. 29, 1797–1805. doi: 10.1007/s00192-018-3767-3 30267143 PMC6527134

[B9] Thomas-White K. J. Hilt E. E. Fok C. Pearce M. M. Mueller E. R. Kliethermes S.. (2016). Incontinence Medication Response Relates to the Female Urinary Microbiota. Int. Urogynecol. J. 27, 723–733. doi: 10.1007/s00192-015-2847-x 26423260 PMC5119460

[B10] Wilkins E. G. Payne S. R. Pead P. J. Moss S. T. Maskell R. M. (1989). Interstitial Cystitis and the Urethral Syndrome: A Possible Answer. Br. J. Urol. 64, 39–44. doi: 10.1111/j.1464-410X.1989.tb05519.x 2670041

