# Supplementary material for: The Urobiomes of Adult Women With Various Lower Urinary Tract Symptoms Status Differ: A Re-Analysis
Source: Front Cell Infect Microbiol. 2022 Jun 9;12:860408. doi: 10.3389/fcimb.2022.860408 (PMC9218574; doi:10.3389/fcimb.2022.860408)
Supplement: Supplemental Table 1 — Source of participants for this pooled analysis. (1) 111 peri-menopausal women with UUI prior to treatment with solifenacin or asymptomatic controls, (2) 154 women with UTI-like symptoms or asymptomatic controls, (3) 134 peri-menopausal asymptomatic controls, (4) 89 women with IC/PBS or asymptomatic controls, (5) 145 women with SUI, UUI, or asymptomatic controls, (6) 63 postmenopausal women with UUI prior to receipt of vaginal estrogen therapy, (7) 225 women with symptoms of UTI recruited to a trial evaluating the expanded urine culture protocol, and (8) 83 peri-menopausal women with UUI prior to oral treatment with mirabegron. [file DataSheet_1.docx]

**Supplemental Table 1: Studies included**

| **Study** | **Total number of participants** | **Cohorts** | **Citation** |
| --- | --- | --- | --- |
| 1 | 111 | 59 UUI  52 Control | Pearce, M.M., Hilt, E.E., Rosenfeld, A.B., Zilliox, M.J., Thomas-White, K., Fok, C., Kliethermes, S., Schreckenberger, P., Brubaker, L., Gai, X., Wolfe, A.J. 2014. The Female Urinary Microbiome: A Comparison of Women With and Without Urgency Urinary Incontinence. mBio. Jul 8;5(4). pii: e01283-14. doi: 10.1128/mBio.01283-14. PMC4161260  Price TK, Hilt EE, Thomas-White K, Mueller ER, Wolfe AJ, Brubaker L. 2019. The urobiome of continent adult women: a cross-sectional study. British Journal of Obstetrics and Gynecology 127(2):193-201. doi: 10.1111/1471-0528.15920. Epub 2019 Oct 9. PMID:31469215 |
| 2 | 154 | 79 UTI  75 Control | Price, T. K., Dune, T., Hilt, E. E., Thomas-White, K. J., Kliethermes, S., Brincat, C., Brubaker, L., Wolfe, A. J., Mueller, E. R., Schreckenberger, P. 2016. The Clinical Urine Culture: Enhanced Techniques Improve Detection of Clinically Relevant Microorganisms. Journal of Clinical Microbiology. May;54(5):1216-22. PMC4844725  Price TK, Hilt EE, Thomas-White K, Mueller ER, Wolfe AJ, Brubaker L. 2019. The urobiome of continent adult women: a cross-sectional study. British Journal of Obstetrics and Gynecology 127(2):193-201. doi: 10.1111/1471-0528.15920. Epub 2019 Oct 9. PMID:31469215 |
| 3 | 134 | 134 Control | Price TK, Hilt EE, Thomas-White K, Mueller ER, Wolfe AJ, Brubaker L. 2019. The urobiome of continent adult women: a cross-sectional study. British Journal of Obstetrics and Gynecology 127(2):193-201. doi: 10.1111/1471-0528.15920. Epub 2019 Oct 9. PMID:31469215 |
| 4 | 89 | 49 IC/PBS  40 Control | Jacobs KM, Price TK, Thomas-White K, Halverson T, Davies A, Myers DL, Wolfe AJ. Cultivable Bacteria in Urine of Women With Interstitial Cystitis: (Not) What We Expected. Female Pelvic Med Reconstr Surg. 2020 Apr 6. doi: 10.1097/SPV.0000000000000854. [Epub ahead of print] PMID:32265402 |
| 5 | 145 | 50 UUI  50 SUI  45 Control | Price TK, Lin H, Gao X, Thomas-White KJ, Hilt EE, Mueller ER, Wolfe AJ, Dong Q, Brubaker L. Bladder Bacterial Diversity Differs In Continent and Incontinent Women: A Cross-Sectional Study. Am J Obstet Gynecol. 2020 May 4:S0002-9378(20)30512-3. doi: 10.1016/j.ajog.2020.04.033 |
| 6 | 63 | 63 UUI | Thomas-White K, Taege S, Limeira R, Brincat C, Joyce C, Hilt EE, Mac-Daniel L, Radek KA, Brubaker L, Mueller ER, Wolfe AJ. Vaginal estrogen therapy is associated with increased *Lactobacillus* in the urine of postmenopausal women with overactive bladder symptoms. American Journal of Obstetrics and Gynecology. 2020 Nov 1;223(5):727-e1 |
| 7 | 225 | 225 UTI | Barnes HC, Wolff B, Abdul-Rahim O, Harrington A, Hilt EE, Price TK, Halverson T, Hochstedler BR, Pham T, Acevedo-Alvarez M, Joyce C. A Randomized Clinical Trial of Standard versus Expanded Cultures to Diagnose Urinary Tract Infections in Women. The Journal of Urology. 2021 Jun 29:10-97. |
| 8 | 83 | 83 UUI | unpublished |

**Supplemental Table 2: Correlation of Patient Characteristics with Alpha Diversity**

|  | UTI | UUI | SUI | IC/PBS | Control |
| --- | --- | --- | --- | --- | --- |
|  | Rho (95% CI) of diversity measure with age within cohort | | | | |
| Number of genera | -0.15 (-0.25, -0.03) | 0.20 (0.08, 0.31) | -0.09 (-0.36, 0.19) | -0.01 (-0.29, 0.27) | 0.10 (-0.01, 0.20) |
| Pielou’s evenness* | -0.16 (-0.29, -0.03) | 0.11 (-0.02, 0.24) | 0.20 (-0.14, 0.49) | -0.01 (-0.34, 0.32) | 0.06 (-0.07, 0.18) |
| Shannon index | -0.16 (-0.27, -0.05) | 0.20 (0.08, 0.32) | 0.11 (-0.17, 0.38) | 0.01 (-0.27, 0.29) | 0.07 (-0.03, 0.18) |
| Simpson index | -0.14 (-0.25, -0.03) | 0.17 (0.05, 0.29) | 0.25 (-0.04, 0.49) | 0.01 (-0.27, 0.29) | -0.05 (-0.15, 0.06) |
|  | Rho (95% CI) of diversity measure with body mass index (kg/m^2^) within cohort | | | | |
| Number of genera | 0.25 (0.14, 0.35) | 0.13 (0.01, 0.25) | 0.21 (-0.08, 0.46) | -- | 0.12 (0.00, 0.22) |
| Pielou’s evenness* | 0.08 (-0.06, 0.21) | -0.11 (-0.23, 0.02) | -0.13 (-0.44, 0.20) | -- | 0.10 (-0.02, 0.23) |
| Shannon index | 0.23 (0.12, 0.34) | 0.02 (-0.10, 0.15) | 0.05 (-0.23, 0.32) | -- | 0.08 (-0.04, 0.19) |
| Simpson index | 0.19 (0.08, 0.30) | -0.10 (-0.22, 0.02) | 0.06 (-0.23, 0.33) | -- | -0.09 (-0.20, 0.02) |

*calculated among those with at least two genera
